# Decoupling optical function and geometrical form using conformal flexible dielectric metasurfaces

**DOI:** 10.1038/ncomms11618

**Published:** 2016-05-19

**Authors:** Seyedeh Mahsa Kamali, Amir Arbabi, Ehsan Arbabi, Yu Horie, Andrei Faraon

**Affiliations:** 1 T. J. Watson Laboratory of Applied Physics, Kavli Nanoscience Institute, California Institute of Technology, 1200 E California Boulevard, Pasadena, California 91125, USA

## Abstract

Physical geometry and optical properties of objects are correlated: cylinders focus light to a line, spheres to a point and arbitrarily shaped objects introduce optical aberrations. Multi-functional components with decoupled geometrical form and optical function are needed when specific optical functionalities must be provided while the shapes are dictated by other considerations like ergonomics, aerodynamics or aesthetics. Here we demonstrate an approach for decoupling optical properties of objects from their physical shape using thin and flexible dielectric metasurfaces which conform to objects' surface and change their optical properties. The conformal metasurfaces are composed of silicon nano-posts embedded in a polymer substrate that locally modify near-infrared (*λ*=915 nm) optical wavefronts. As proof of concept, we show that cylindrical lenses covered with metasurfaces can be transformed to function as aspherical lenses focusing light to a point. The conformal metasurface concept is highly versatile for developing arbitrarily shaped multi-functional optical devices.

## Abstract (web summary)

Realizing components with decoupled geometrical form and optical function are of interest for various applications. Here, Kamali *et al*. introduce flexible metasurfaces based on a transmitarray platform that can be conformed to a non-planar arbitrarily shaped object to modify its optical properties at will.

## Graphical abstract

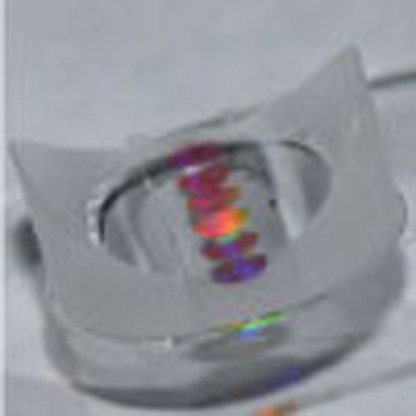

The correlation between the geometry of an object and its optical functionality^1^ has introduced long-standing design challenges to optical engineers developing multi-functional components^2^. The traditional solution has been to compromise and optimize the component material and geometry by considering all the physical requirements. This was originally studied in the context of conformal and freeform optics where optical components with non-standard surfaces were developed for integration of optics into flying objects with specific aerodynamic shapes^3^,^4^. More recently, this issue has attracted new attention due to its application in integration of optics into various consumer electronic products and medical equipment with stringent packaging and design requirements. Furthermore, controlling optical properties of objects without physically modifying them can enable the visual blending of an object with its background^5^,^6^,^7^,^8^ or changing its appearance through generation of a holographic virtual image^9^,^10^. In the context of conformal optics, the conventional solution is to stack several bulky optical elements with non-standard surface profiles underneath the outermost surface of the object^4^. Such solutions usually have challenging fabrication processes requiring custom-made fabrication equipment, are bulky and do not provide a unified and versatile approach that can be applied to arbitrary geometries. Conformal metasurface approach can provide a solution for decoupling the geometric shape and optical characteristics of arbitrary objects.

Metasurfaces are two-dimensional (2D) arrays of scatterers rationally designed to locally modify phase and polarization of electromagnetic waves^11^,^12^,^13^,^14^. They enable wafer-scale production of lithographically defined thin diffractive optical elements using conventional nano-manufacturing techniques. These manufacturing techniques are optimized for patterning flat substrates and are not applicable for the direct fabrication of metasurfaces on non-planar structures required for conformal optics. However, the 2D nature and the minute thickness of optical metasurfaces make them suitable for transferring to non-planar substrates. Several different plasmonic and dielectric metasurface platforms for optical wavefront manipulation have been recently proposed^11^,^12^,^13^,^14^,^15^,^16^,^17^,^18^. Among different platforms, dielectric metasurfaces based on high-contrast transmitarrays are highly versatile^14^,^17^,^18^ as they provide simultaneous manipulation of phase and polarization of light with high efficiencies, and can sample optical wavefronts with subwavelength spatial resolution^14^. In these metasurfaces, each meta-atom is a high-index nano-post acting as a short waveguide, which locally imposes a certain phase shift and polarization rotation. Several efforts have been made to transfer metasurfaces (mostly plasmonic ones) to flexible substrates with the aim of tuning their frequency response using substrate deformation^19^,^20^,^21^,^22^,^23^,^24^,^25^. Plasmonic metasurfaces, however, have low efficiencies, especially in the transmission mode, which in many situations make them impractical.

Here, we introduce flexible metasurfaces based on a dielectric high-contrast transmitarray platform that can be conformed to a non-planar arbitrarily shaped object to modify its optical properties at will. We present a general design procedure and a high-yield fabrication process for the conformal flexible metasurface platform. As proof of principle, we experimentally demonstrate flexible metasurfaces that wrap over cylindrical surfaces and convert them to aspherical lenses.

## Results

### Conformal metasurfaces platform

**Figure 1:**
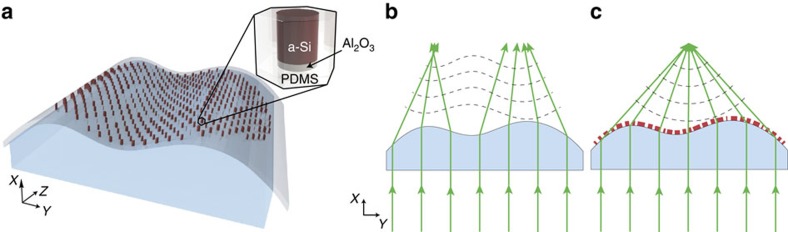
Conformal optics with optical dielectric metasurfaces. (**a**) A schematic illustration of a dielectric metasurface layer conformed to the surface of a transparent object with arbitrary geometry. (Inset) The building block of the metasurface structure: an amorphous silicon (a-Si) nano-post on a thin layer of aluminium oxide (Al_2_O_3_) embedded in a low-index flexible substrate (PDMS for instance). (**b**) Side view of the arbitrarily shaped object showing how the object refracts light according to its geometry and generates an undesirable wavefront. (**c**) The same object with a thin dielectric metasurface layer conformed to its surface to change its optical response to a desired one.

Figure 1a shows a schematic illustration of a non-planar arbitrarily shaped transparent object wrapped by a flexible metasurface based on this platform. The metasurface layer is composed of an array of dissimilar cylindrical amorphous silicon (a-Si) nano-posts with different diameters placed on a subwavelength periodic hexagonal lattice and embedded in polydimethylsiloxane (PDMS) as a flexible substrate (Fig. 1a, inset). The arbitrary shape of the object's surface distorts the wavefront of the transmitted light in an undesirable way (Fig. 1b). By conforming the metasurface onto the object's outermost surface, the distortion can be compensated and the wavefront of the transmitted light can be shaped to a desired form, similar to phase-compensating antenna arrays used in the microwave regime^26^. For example, the metasurface can be designed to correct the distortions introduced by the arbitrarily shaped object and make it act similar to an aspherical lens that focuses light to a point as schematically shown in Fig. 1c.

### Operation principle and design procedure

The desired phase profile of the conformal metasurface is found with the knowledge of the geometry of the transparent object over which it is wrapped, and the desired optical response. First, the object without the metasurface is considered, and the phase profile of the optical waves transmitted through the object is computed along the surface of the object. For objects with dimensions significantly larger than the optical wavelengths, this phase profile can be found using ray optics approximation and by computing the optical path length and the corresponding optical path difference (OPD) of the rays passing through different points along the outermost surface of the object with respect to the chief ray. Then, using a similar OPD-based approach, the phase profile required to achieve the desired specific functionality is obtained along the surface of the object. For example, if we want the object to focus light to a point, a converging spherical wavefront is desired, which is sampled along the arbitrary surface of the object. The metasurface layer, when wrapped on the surface of the object, should locally impose an additional optical phase shift equal to the difference between the original phase of the object and the desired phase profile. Therefore, the desired metasurface phase profile is expressed as a function of two coordinate values defining the non-planar surface of the object. To obtain the appropriate phase profile of the metasurface before its transfer to the non-planar surface, an appropriate coordinate transformation should be applied. For example, if the flexible substrate of the metasurface is under no stress after being mounted on the object's surface, then the appropriate coordinate transformation conserves length along the surface of the object.

**Figure 2:**
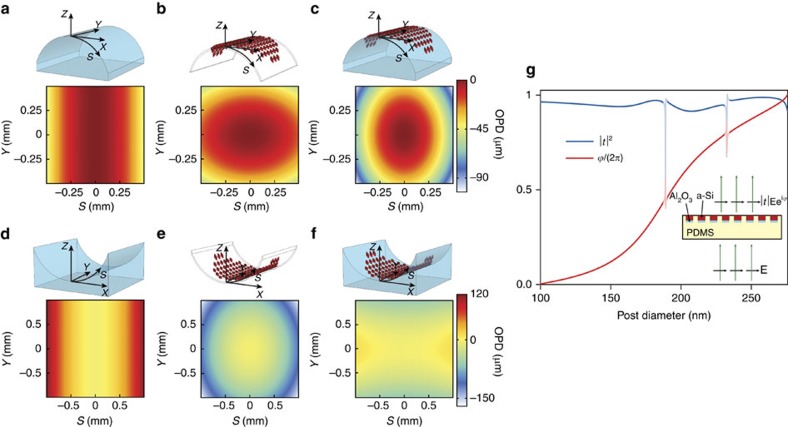
Design procedure of conformal metasurfaces. (**a**) The OPD (in μm) of the rays passing through a converging cylindrical object. (**b**) The difference OPD needed at the surface of the convex cylindrical object compensated by the conformal metasurface. (**c**) Desired OPD at the surface of the object which is provided by the object and conformal metasurface combination. (**d**–**f**) show similar plots for a diverging cylinder. ‘*S*' is the arch length on the cylinder surface in a plane perpendicular to the *y*-axis. (**g**) Simulated intensity transmission and phase of the transmission coefficient for a periodic array of amorphous silicon (a-Si) nano-posts embedded in PDMS as shown in the inset. The nano-posts are composed of 720 nm a-Si on 100 nm aluminium oxide (Al_2_O_3_), and are arranged in a hexagonal lattice. The simulation wavelength is 915 nm. This graph is used to relate the phase-shift values (and the respective OPDs) needed at different points on the conformal metasurface to the nano-post diameters. See the ‘Methods' section for simulation details.

Using this design procedure, we computed two sets of conformal metasurface phase profiles for both a convex and a concave cylindrical glass. The metasurfaces modify the wavefronts of the cylindrical objects to make them behave as aspherical lenses. Figures 2a-2d show the OPD of the rays passing through the convex and concave cylinders at their top surfaces, respectively. Considering the desired converging spherical wavefronts, the desired OPDs of the rays at the surfaces of the convex and concave cylinders are calculated and shown in Figs 2c-2f, respectively. The differences between the OPDs of the convex and concave cylindrical objects and their corresponding converging spherical phase profiles are shown in Figs 2b-2e, respectively. The conformal metasurfaces should impose phase shifts equivalent to these OPDs at the operation wavelength (see the ‘Methods' section for simulation details). Since the cylindrical surfaces are isometric with a plane, the metasurfaces can be mounted on them under negligible stress. Therefore, only a simple geometric transformation (*XY* to *SY* in Fig. 2a) is used to map the coordinates on a cylinder surface to a plane.

The optical coupling among the nano-posts is weak in the high-contrast transmitarray metasurface platform, and each nano-post scatters light almost independent of its neighbouring nano-posts. The weak coupling is due to the high-index contrast between the nano-posts and their surroundings, and it is manifested in the localization of the optical energy inside the nano-posts and the weak dependence of the transmission of the nano-post arrays to their spacing (that is, lattice constant) as has been previously discussed^17^ in more detail. This simplifies the design by allowing to directly relate the local transmission coefficient to the diameter of the nano-post at each unit cell of the metasurface. Figure 2g shows the simulated intensity transmission coefficient and phase of the transmission coefficient for periodic arrays of 720-nm tall nano-posts embedded in PDMS with diameters ranging from 100 to 275 nm (see the ‘Methods' section for simulation details). The nano-posts are arranged in a hexagonal lattice with 550 nm lattice constant, and the simulation wavelength is 915 nm. Refractive indices of a-Si and PDMS are 3.56 and 1.41 at the simulation wavelength, respectively. The whole 0 to 2*π*-phase range can be covered by changing the nano-post diameters while keeping the intensity transmission coefficient above 91%. These results are obtained assuming normal incidence.

To get more insight into the operation mechanism, each nano-post can be considered as a truncated circular cross-sectional waveguide^27^. Because of the truncation of both ends, the nano-post supports multiple low-quality factor Fabry–Perot resonances which interfere and lead to high transmission of the nano-post array (see Supplementary Note 1 and Supplementary Fig. 1). We also note that in contrast to Huygens' metasurfaces, where only two resonant modes are used (one with a significant electric dipole and one with significant magnetic dipole)^28^, the resonant modes of the nano-posts contain dipole, quadrupole and higher-order electric and magnetic multipoles in their multipole expansion. Although the modal expansion approach provides some intuitive understanding of the operation principle, it does not offer guidelines for designing of the nano-post arrays. Moreover, an effective medium method does not capture the underlying physics of the periodic structures that support more than one propagating mode^18^,^27^,^29^. Therefore it is not applicable to most of the nano-posts widths we used in designing the metasurface, because a periodic array of nano-posts with diameters >180 nm would be multimode. Considering these, and the limited number of design parameters (that is, nano-post height and the lattice constant), we prefer the direct approach of finding the transmission of the nano-post arrays (as shown in Fig. 2g) over the modal expansion technique.

Low sensitivity to the incident angle is a necessary property for a conformal metasurface since the incident angle would be varying across the metasurface when it is wrapped over a non-planar object. For the metasurface platform considered here, the transmission coefficient of transverse electric (TE) polarized light is weakly dependent on the incidence angle, and transmission coefficient of transverse magnetic (TM) polarized light shows some angle-dependent resonances (Supplementary Note 2 and Supplementary Fig. 2). These resonances introduce a small-phase error and lower transmission, but as we experimentally show, they only slightly reduce the metasurface efficiency for TM polarization. For very steep angles, the metasurface efficiency decreases as analysed in our previous work^17^. The general metasurface design procedure is as follows. First, the coordinate-transformed desired metasurface phase was sampled at the lattice sites of the periodic hexagonal lattice. Then, the diameter of the nano-post at each site was obtained using the corresponding sampled phase value at that site and the phase-diameter relation shown in Fig. 2g. To ensure a one-to-one relationship between the phase and nano-post diameters, and to keep the transmission high, nano-post diameters corresponding to the sharp resonances in Fig. 2g were not used. Using this procedure, metasurfaces with phase profiles shown in Figs 2b-2e were designed to be conformed to convex and concave cylindrical objects, respectively. These metasurfaces modify the optical response of the cylinders such that they behave as aspherical lenses and focus light to single points (see the ‘Methods' section for the details of designed lenses and cylindrical surfaces).

### Fabrication and characterization of conformal metasurfaces

**Figure 3:**
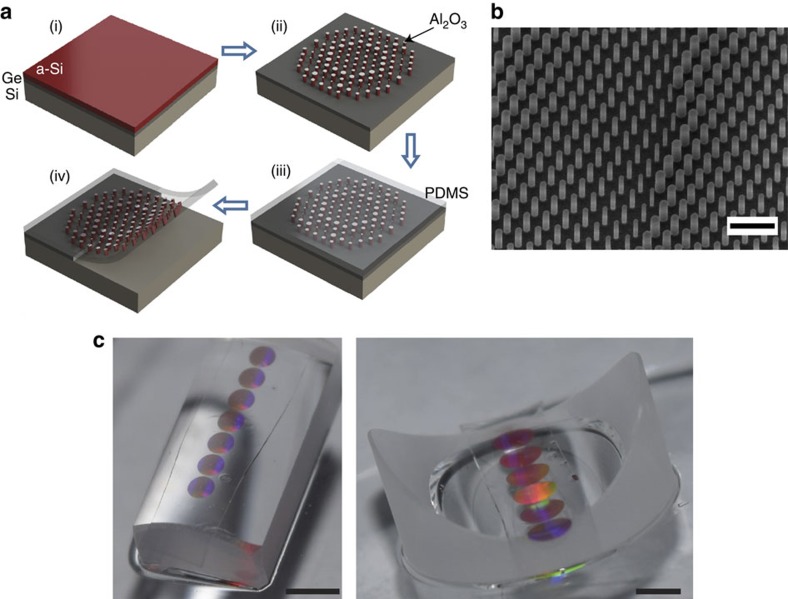
Overview of the fabrication process and images of the fabricated metasurfaces. (**a**) Steps involved in the fabrication of conformal metasurfaces: (i) Germanium (Ge) and amorphous silicon (a-Si) are deposited on a silicon wafer. (ii) a-Si nano-posts are patterned and dry etched using an aluminium oxide hard mask. (iii) PDMS is spin coated on the substrate. (iv) The sacrificial Ge layer is dissolved to release the nano-posts which are embedded in the flexible PDMS layer. (**b**) A scanning electron microscope image of the silicon nano-posts with the aluminium oxide mask before spin coating PDMS. Scale bar, 1 μm. (**c**) Optical images of two flexible metasurfaces conformed to a convex glass cylinder (left) and a concave glass cylinder (right). In both cases, the metasurfaces make cylinders behave like converging aspherical lenses. Scale bar, 2 mm.

**Figure 4:**
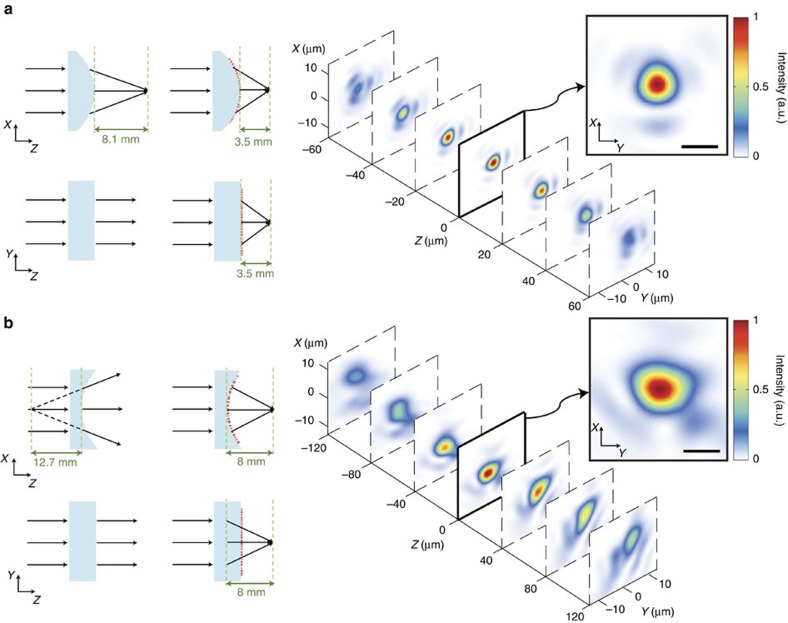
Measurement results of conformal dielectric metasurfaces. (**a**) A converging cylindrical lens with a radius of 4.13 mm and a focal distance of 8.1 mm is optically modified using a conformal metasurface with a diameter of 1 mm. The cylinder plus the metasurface combination behaves as an aspherical lens with a focal length of 3.5 mm. The coordinate system is the same as in Fig. 2. (**b**) A different metasurface is mounted on a concave glass cylinder with a radius of 6.48 mm and a focal distance of −12.7 mm, which makes it focus to a spot 8 mm away from its surface (as an aspherical lens). Schematic illustrations (side and top views) are shown on the left, and intensities at planes parallel to the focal plane and at different distances from it are shown on the right. Intensities at the focal plane are depicted in the insets. Measurements are performed at the wavelength of 915 nm. For the measurement details see the ‘Methods' section. Scale bar, 5 μm.

Figure 3a schematically illustrates the key steps in fabricating thin, flexible and conformable metasurfaces. A germanium sacrificial layer is deposited on a silicon wafer and then an a-Si layer is deposited over the germanium (Fig. 3a (i)). The a-Si layer is patterned using electron-beam lithography followed by dry etching using an alumina hard mask (Fig. 3a (ii)). The sample is subsequently spin coated with two layers of PDMS (a diluted thin layer followed by a thicker layer (Fig. 3a (iii)). Then, the sample is immersed in a diluted ammonia solution which dissolves the germanium layer and releases the flexible metasurface with minimal degradation of the metasurface and the PDMS layer (Fig. 3a (iv)). A scanning electron microscope image of the fabricated device before spin coating the PDMS layer is shown in Fig. 3b. Optical images of metasurfaces conformed to the convex and concave glass cylinders are shown in Fig. 3c. The whole fabrication process has a near-unity yield, with almost all of the metasurfaces retaining a large majority of the nano-posts (Supplementary Note 3 and Supplementary Fig. 3). Moreover, it does not degrade the optical quality of the metasurface layer. The optical quality of the flexible metasurface layer was tested by transferring a flat metasurface lens to a flat substrate. See Supplementary Fig. 4 for the measurement results and focusing efficiency of the transferred flat metasurface lens. To demonstrate the capabilities of this platform, two different conformal metasurfaces operating at the near-infrared wavelength of 915 nm were fabricated and characterized. The first 1-mm-diameter metasurface conforms to a converging cylindrical lens with a radius of 4.13 mm. The cylinder by itself focuses light to a line 8.1 mm away from its surface (Fig. 4a). The presence of the metasurface modifies the cylinder to behave as an aspherical lens focusing light to a point 3.5 mm away from the surface of the cylinder (Fig. 4a). The second device is a 2-mm-diameter metasurface conforming to a diverging glass cylinder with a radius of 6.48 mm and a focal length of −12.7 mm (Fig. 4b). With the metasurface on top, the concave cylinder focuses light to a point 8 mm away from the cylinder surface (Fig. 4b).

The devices were characterized under 915 nm collimated laser beam illumination by recording intensity profiles at different planes parallel to their focal planes. Figure 4 also shows the measured intensity profiles. The focal-plane intensity profiles are shown as insets. A tight focus is observed at the designed focal length. Focusing efficiencies of 56 and 52% under TE illumination (that is, electrical field parallel to the cylinder axis) were measured for the two devices, respectively. The focusing efficiency is defined as the ratio of the power focused by the device to the incident power on the device (see the ‘Methods' section for the measurement details). Under TM illumination, numerical estimations based on the angular response of a uniform array shown in Supplementary Fig. 2 indicate a slight degradation of the device performance for larger angles between the metasurface and the incident beam. The devices were measured with TM input beam polarizations and, as expected, showed similar behaviour as under TE illumination with focusing efficiencies of 56 and 50%. The difference in TE and TM polarization efficiencies increases as the incidence angle becomes steeper (Supplementary Fig. 5); the focus pattern, however, remains almost the same under both polarizations (Supplementary Fig. 6). The corresponding measured full width at half maximum (FWHM) of the focal spots are ∼3.5 and 5 μm and are comparable to the diffraction-limited FWHM of 3.2 and 3.7 μm, respectively. Slight aberrations observed in the focal-plane intensity profiles are mostly due to imperfections in the alignment of the metasurface to the non-planar substrates. Reduction of efficiency in conformal metasurfaces compared with the transferred flat metasurfaces (Supplementary Fig. 4) is mostly due to the imperfections in the alignment, slight movements of the nano-posts within the flexible substrate during the substrate handling, and the difference between the actual non-planar substrate profile and the profile assumed for design.

## Discussion

Although here we have used cylindrical substrates as proof of principle, this platform is not limited to surfaces that can be projected to a plane using isometric transformations. Conformal metasurfaces can be designed for other types of objects (for instance spheres where the metasurface needs to be stretched for conforming) with a similar method. High stretchability and flexibility of thin PDMS layers (∼50 μm) make them suitable for conforming to non-isometric surfaces. In such cases, however, a mechanical analysis of the metasurface deformation upon mounting on the object should be carried out. The coordinate transformation that projects the conformal lattice to the planar one should also account for this deformation. Besides, in the case of objects with steep angles (where the incident collimated beam is far from normal to the metasurface at some points), further considerations should be taken in choosing the lattice constant to avoid excitation of higher-order diffractions. Moreover, since the design procedure is local (that is, each nano-post at each lattice site is chosen independently), the incident angle of the beam at each lattice point can be taken into account in designing the respective nano-post.

Conformal dielectric metasurfaces operate based on spatially varying nano-structured diffractive scatterers. The behaviour of the device is wavelength dependent because both the optical response of the scatterers and their arrangement is optimized for a given wavelength. The performance of the proposed devices has a wavelength dependence similar to other high-contrast transmitarray lenses recently demonstrated^17^, where good performance is maintained over a bandwidth of a few per cent around the design wavelength.

The proposed platform is relatively robust to systematic and random errors. Fabrication errors do not affect the device functionality and only reduce its efficiency (5 nm error in nano-post diameters results in ∼3% reduction of efficiency^14^). Alignment imperfections (extra stretch or angular rotation) results in focal distance mismatch between the non-planar object and the metasurface. Microlens focal distance has second-order dependence on the substrate stretch ratio. For instance, for the devices shown in Fig. 4, having 1% strain in the flexible metasurface results in a 2% error in focal distance and a 1 degree rotation misalignment results in 0.06% mismatch between the horizontal and vertical focal distances. Also, fractional wavelength error is equal to the fractional error of the focal distance^17^ (that is, 1% error in wavelength results in 1% error in the focal distance of the flexible metasurface).

The developed fabrication process has a near-unity yield and we are able to transfer almost all (larger than 99.5%) of the nano-posts into the PDMS with good accuracy. Nevertheless, the proposed platform is very robust to the fabrication deficiencies; various imperfections including deviations between designed and fabricated nano-post sizes (∼5 nm in the diameter and (or) height of the nano-posts), rough side walls, and missing nano-posts only result in small reductions in the efficiency of the device, and does not alter the functionality significantly.

In conclusion, we demonstrated flexible dielectric metasurfaces and showed their applications for conformal optics. As proof of concept, the optical properties of glass cylinders have been changed to behave like aspherical lenses focusing light to a point. The design paradigm can be applied to any other system where conformal optical design is required. In addition, flexible electronics is a well-established field of research, with the aim of transferring conventional systems to flexible and non-planar substrates. Very promising results have been achieved during the last decade with various applications in wearable electronics, electronic skins and medical devices^30^,^31^,^32^. The flexible and conformal metasurface platform proposed here can be merged with conformal electronics leading to versatile flexible optoelectronic technologies.

## Methods

### Design procedure

The optical path length and the corresponding OPD of light passing through the cylinders were computed using the ray optics approximation. For simulations, the convex and concave cylinders were assumed to have radii of 4.13 and 6.48 mm, respectively, and a refractive index of 1.507. The PDMS layer was modelled as a 50-μm-thick layer with a refractive index of 1.41. In both cases, the object OPDs were calculated at the outermost surface of the PDMS, considering light propagation through the PDMS layer and refraction at the glass-PDMS interface. The desired OPDs were also calculated at the same surfaces, assuming focal distances of 3.5 and 8 mm for the convex and concave lenses, respectively. Two different metasurfaces of diameters 1 and 2 mm were designed for the convex and concave cylinders to impose the phase shifts equivalent to the difference of the cylinders' and the desired OPDs.

The planar periodic metasurfaces were simulated using the rigorous coupled wave analysis technique to find the complex transmission coefficients corresponding to all nano-post diameters for normal incident plane waves (Fig. 2g)^33^. The lattice constant is chosen such that the array is non-diffractive at the simulation wavelength. Simulation results shown in Supplementary Fig. 2 were also obtained using the rigorous coupled wave analysis technique. All of the simulations and calculations were performed at the wavelength of 915 nm.

### Sample fabrication

A 300-nm-thick germanium sacrificial layer was deposited by electron-beam evaporation on a silicon wafer, and 720 nm hydrogenated a-Si was deposited on the germanium layer using plasma-enhanced chemical vapour deposition with a 5% mixture of silane in argon at 200 °C. The refractive index of the a-Si layer was measured using variable-angle spectroscopic ellipsometry and was found to be 3.56 at the wavelength of 915 nm. The metasurface pattern was defined in ZEP-520A positive resist (∼300 nm, spin coated at 5,000 r.p.m. for 1 min) using a Vistec EBPG5000+ electron-beam lithography system. The pattern was developed in a resist developer (ZED-N50 from Zeon Chemicals). After developing the resist, the pattern was transferred into a ∼100-nm-thick aluminium oxide layer deposited by electron-beam evaporation through a lift-off process. The patterned aluminium oxide served as a hard mask for dry etching of the a-Si layer in a mixture of SF_6_ and C_4_F_8_ plasma. The PDMS polymer (RTV-615 A and B mixed with a 10:1 mass ratio) was diluted in toluene in a 2:3 weight ratio as a thinner. The mixture was spin coated at 3,000 r.p.m. for 1 min on the fabricated metasurface to fill the gaps between the nano-posts and to form a thin PDMS film (Supplementary Fig. 3). The sample was degassed and cured for >30 min. The second layer of PDMS without a thinner was spin coated on the sample to form a ∼50-μm-thick PDMS film (spin coated at 1,000 r.p.m. for 1 min). The sample was degassed and cured for >1 h. Finally, immersion in a 1:1:30 mixture of ammonium hydroxide, hydrogen peroxide and deionized water at room temperature removed the sacrificial germanium layer releasing the PDMS substrate and the embedded nano-posts (∼1 day). The released metasurface is then mounted manually on the cylinders (Edmund Optics 43–856 and 47–748). To compensate for the misalignment of the substrate and metasurface, multiple lenses with slightly different rotations were fabricated in each sample (Fig. 3c). This way, the best-aligned microlens should have a rotation error of less than or equal to one degree (the rotation step between two successive metasurface lenses).

### Measurement procedure

Devices were characterized using the setups shown schematically in Supplementary Fig. 7. A 915-nm fibre-coupled semiconductor laser was used as the source and a fibre collimation package (Thorlabs F220APC-780) was used to collimate the beam. Intensity at different planes was captured by using a × 50 objective lens (Olympus LMPlanFL N, NA=0.5), a tube lens (Thorlabs LB1945-B) with focal distance of 20 cm, and a camera (CoolSNAP K4 from Photometrics) as shown in Supplementary Fig. 7a. Moreover, neutral density filters (Thorlabs ND filters, B coated) were used to adjust the light intensity and decrease the background noise captured by the camera. The overall microscope magnification was measured by imaging a calibration sample with known feature sizes. To measure the efficiencies, an additional lens (Thorlabs LB1945-B with focal length of 20 cm) was used to partially focus the collimated beam, so that >99% of the beam power falls inside the device under test. The beam radius was adjusted by changing the distance between the lens and the sample. A 15-μm-diameter pinhole (approximately three times the measured FWHM) was placed at the focal plane of the sample to only allow the light focused inside the pinhole area to pass through. The focusing efficiency was then determined as the ratio of measured optical power after the pinhole (that is, the power in focus) to the measured power right before the sample (the incident power). The measurement setup used for efficiency characterization is shown in Supplementary Fig. 7b. For polarization sensitivity measurement, a polarizer (Thorlabs LPNIR050-MP) was added before the sample to set the polarization state of the incident beam.

The data that support the findings of this study are available from the corresponding author on request.

## Additional information

**How to cite this article:** Kamali, S. M. *et al*. Decoupling optical function and geometrical form using conformal flexible dielectric metasurfaces. *Nat. Commun.* 7:11618 doi: 10.1038/ncomms11618 (2016).

## Supplementary Material

Supplementary Information Supplementary Figure 1-7, Supplementary Note 1-3 and Supplementary Reference
