# Supplementary material for: Decoupling optical function and geometrical form using conformal flexible dielectric metasurfaces
Source: Nat Commun. 2016 May 19;7:11618. doi: 10.1038/ncomms11618 (PMC4874029; doi:10.1038/ncomms11618)
Supplement: Supplementary Information — Supplementary Figure 1-7, Supplementary Note 1-3 and Supplementary Reference [file ncomms11618-s1.pdf]

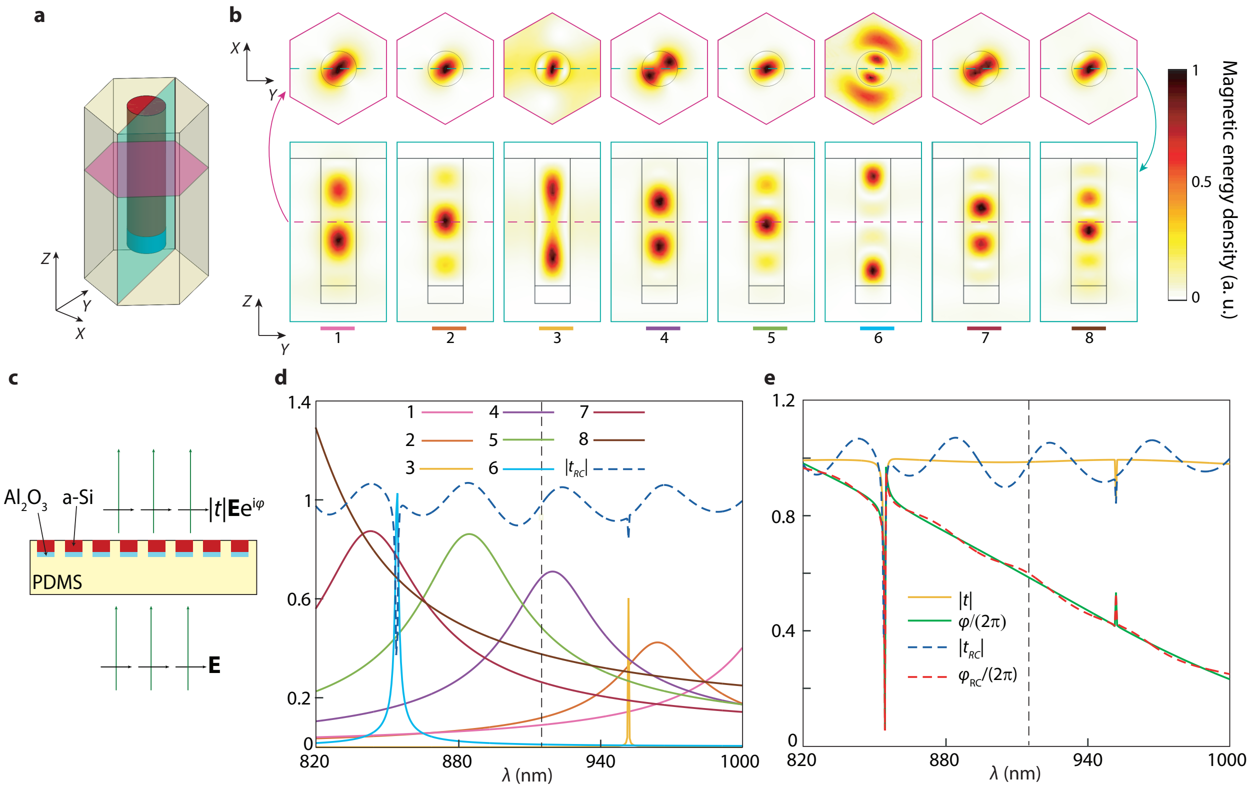

Supplementary Figure 1. **Resonant modes of the nano-posts and their contribution to transmission.** **(a)** Schematic illustration of the metasurface unit cell. **(b)** Magnetic energy density distribution of 8 dominant resonant modes in the bandwidth from 820 nm to 1000 nm, at horizontal (top) and vertical (bottom) cross sections shown in **a**. **(c)** Schematic illustration of a uniform array of nano-posts illuminated with a normally incident plane wave. The amplitude of the transmission coefficient ($|t|$) and its phase (φ) are indicated in the illustration. **(d)** Contribution of 8 dominant resonant modes to the transmission of a periodic array of nano-posts with diameter of 200 nm, as well as the reconstructed transmission amplitude from these 8 modes. **(e)** Transmission amplitude and phase of the periodic array of nano-posts, and reconstructed transmission amplitude and phase using the 8 dominant resonant modes. $|t_{RC}|$: reconstructed transmission amplitude, $|\phi_{RC}|$: reconstructed transmission phase.

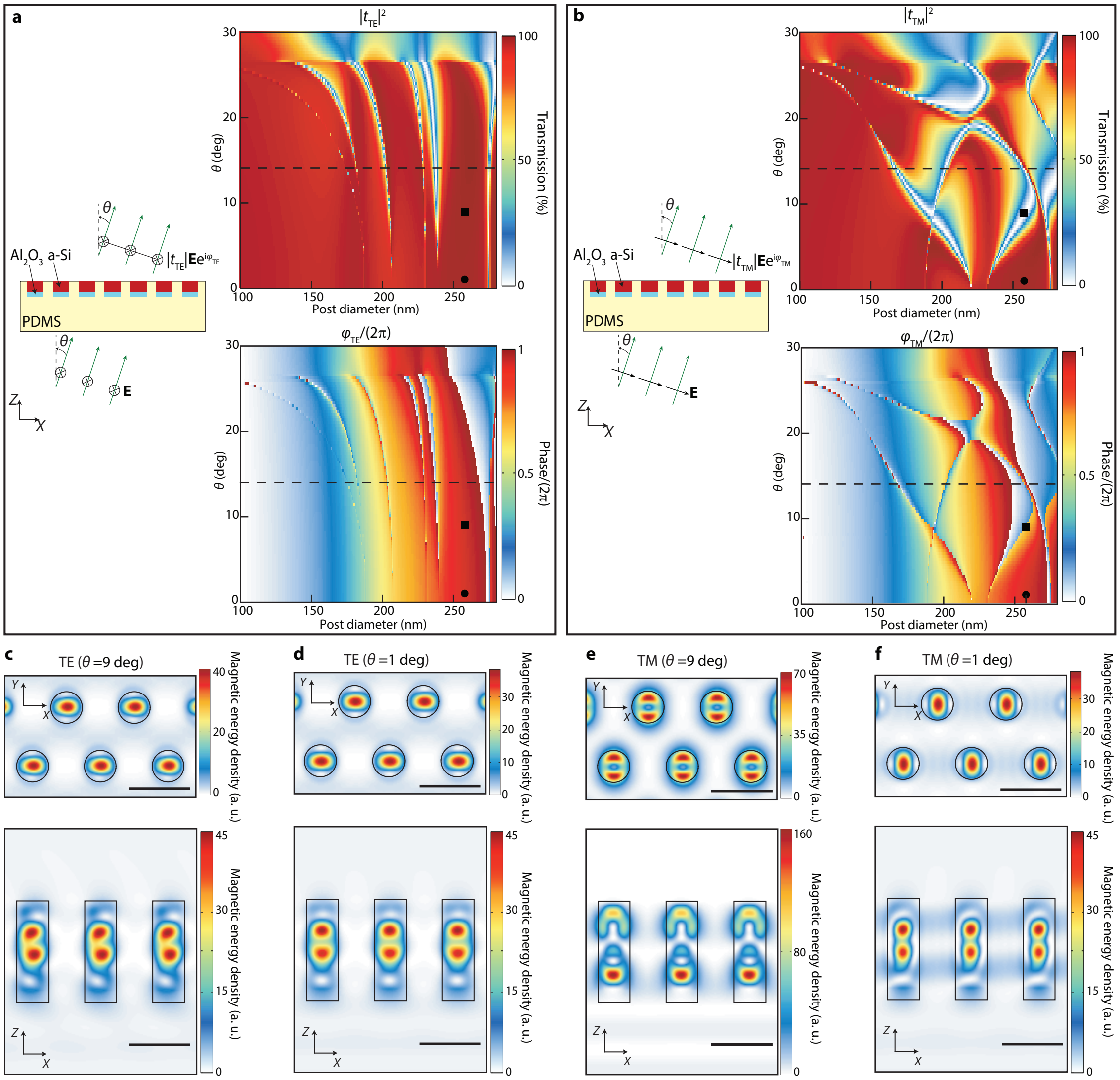

Supplementary Figure 2. **Angular dependence of the transmission coefficient.** **(a)** Schematic illustration of a uniform array of nano-posts embedded in polydimethylsiloxane (PDMS) illuminated by an obliquely incident plane wave (left), and its simulated transmission amplitude and phase as a function of nano-post diameter and incident beam angle for TE and TM (**b**) polarizations. Black dashed lines indicate the largest angle between the metasurface normal and the incident beam used in this manuscript. **(c)** Top and side views of magnetic energy density distribution for TE polarization under an oblique incident plane wave with θ=9°(solid squares in **a**) and **(d)** θ=1°(solid circles in **a**). **(e)** Top and side views of magnetic energy density distribution for TM polarization under an incident plane wave with θ=9° (solid squares in **b**) and **(f)** θ=1° (solid circles in **b**). Black solid lines show the boundaries of the nano-posts. The magnetic energy density of the incident plane wave is chosen to be 1. Scale bars, 500 nm. TE: transverse electric, TM: transverse magnetic.

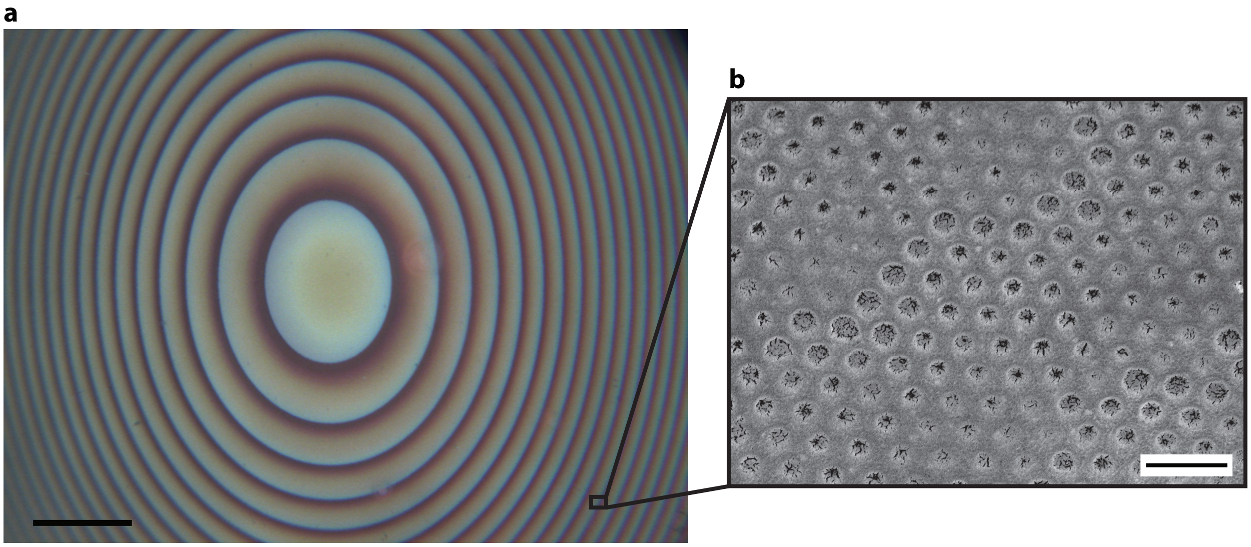

Supplementary Figure 3. **Complete embedding of amorphous silicon nano-posts in PDMS. (a)** Optical microscope image of a portion of a fabricated conformal metasurface lens after transferring to a flexible substrate. This image shows that almost all of the nano-posts are retained in the transfer process. Scale bar, 100 μm. **(b)** Scanning electron microscope image of a portion of the flexible metasurface, taken at a tilt angle of 30 degrees. The image shows silicon nano-posts are entirely embedded in the flexible substrate (polydimethylsiloxane (PDMS)), and void-free filling of the gaps between the nano-posts with PDMS. To dissipate charge accumulation during scanning electron imaging, a $\sim$15-nm-thick gold layer was deposited on the sample prior to imaging. The cracks seen in the gold layer at the position of the nano-posts were not present initially and were gradually formed as the sample was exposed to the electron beam. Scale bar, 1 μm.

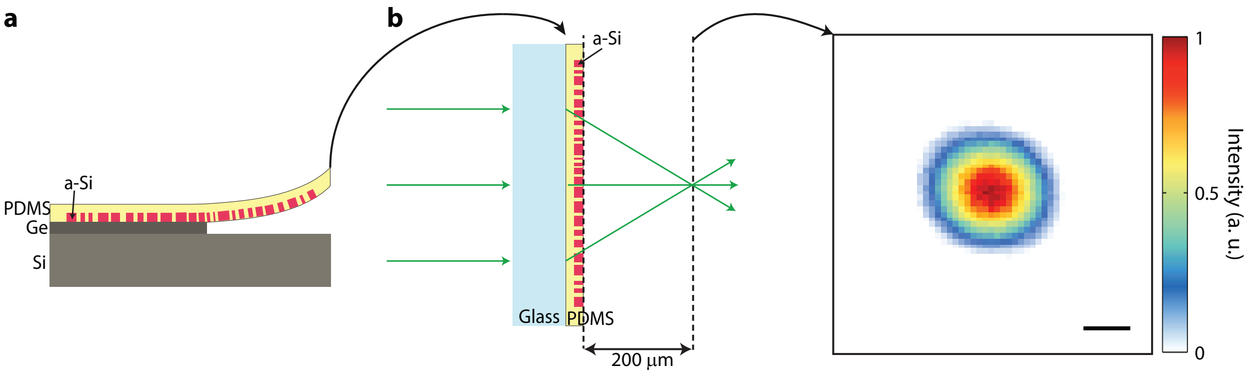

Supplementary Figure 4. **Preservation of high efficiency and diffraction limited optical performance of the metasurfaces through the transfer process.** **(a)** A metasurface lens designed to operate as an aspherical lens when mounted on a flat substrate is transferred to a polydimethylsiloxane (PDMS) substrate. The metasurface lens has a diameter of 200 μm and a focal distance of 200 μm. **(b)** Measured focal plane intensity profile when the flexible metasurface is mounted on a flat glass substrate and illuminated with a collimated beam (as shown in the inset). The measured full width at half maximum (FWHM) spot size of $\sim$ 1 μm agrees well with the diffraction limited FWHM spot size of 1 μm. The focusing efficiency of the lens was measured as 70%. The diffraction limited spot size and the relatively high focusing efficiency verifies the fidelity of the fabrication process in preserving optical properties of metasurfaces. Measurements are performed at the wavelength of 915 nm (see Methods for measurement details). Scale bar, 1 μm.

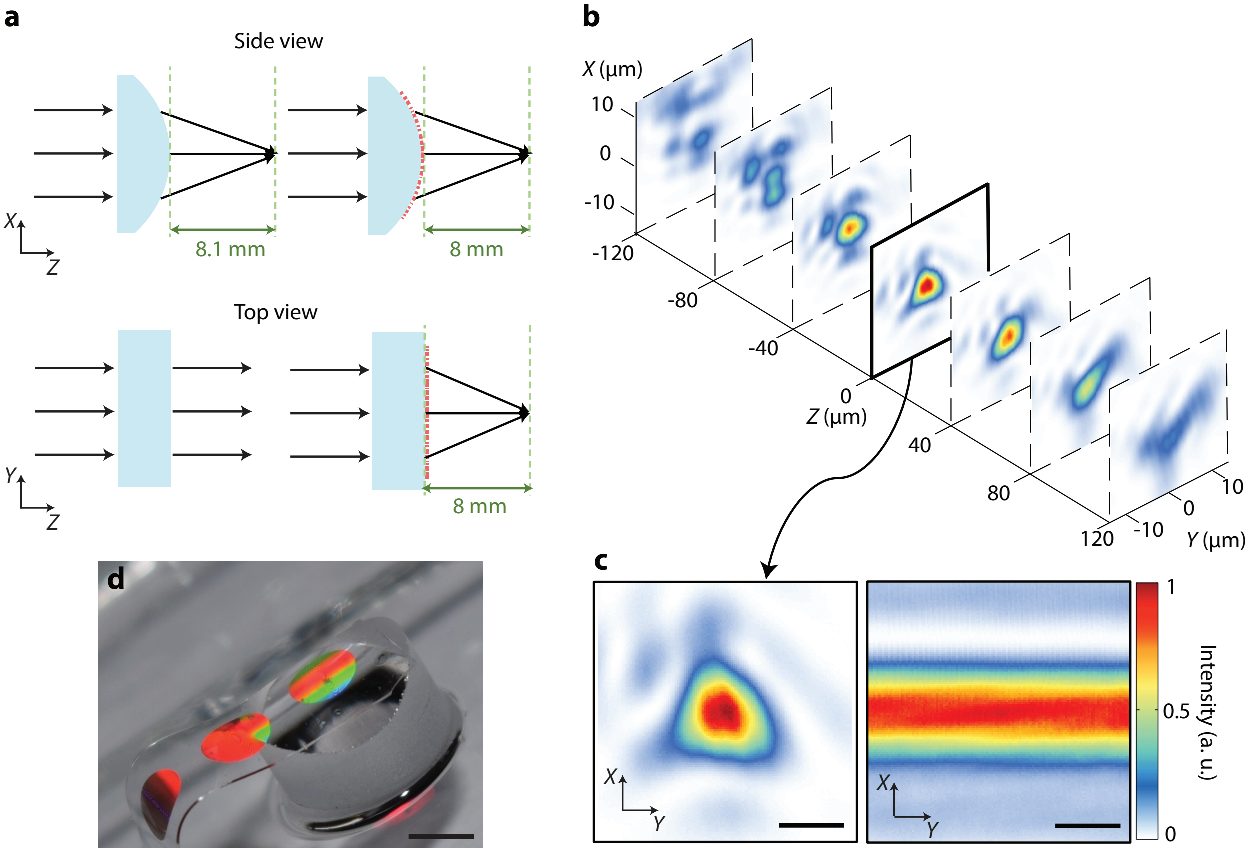

Supplementary Figure 5. **Conformal metasurface with steep incident angles. (a)** Schematic illustration of a metasurface which converts a cylindrical lens to an aspheric lens. The metasurface diameter is 2 mm, and the largest angle between the metasurface normal and the incident beam is 14°. **(b)** Intensities measured at different planes parallel to the focal plane of the cylinder metasurface combination. **(c)** Measured intensities for the glass cylinder (right) and cylinder plus metasurface combination (left) at their respective focal planes. The measured full width at half maximum (FWHM) spot size at the focal plane is approximately 4.5 μm, which is comparable to the diffraction limited FWHM spot size of 3.7 μm. For the metasurface cylinder combination, by using a setup shown in Supplementary Fig. 7b, focusing efficiencies of 68% and 64% were measured for transverse electric (TE) and transverse magnetic (TM) polarizations, respectively. All the measurements are performed at the wavelength of 915 nm. Scale bars, 5 μm. **(d)** Optical image of the fabricated metasurface mounted on the glass cylinder. Scale bar, 2 mm.

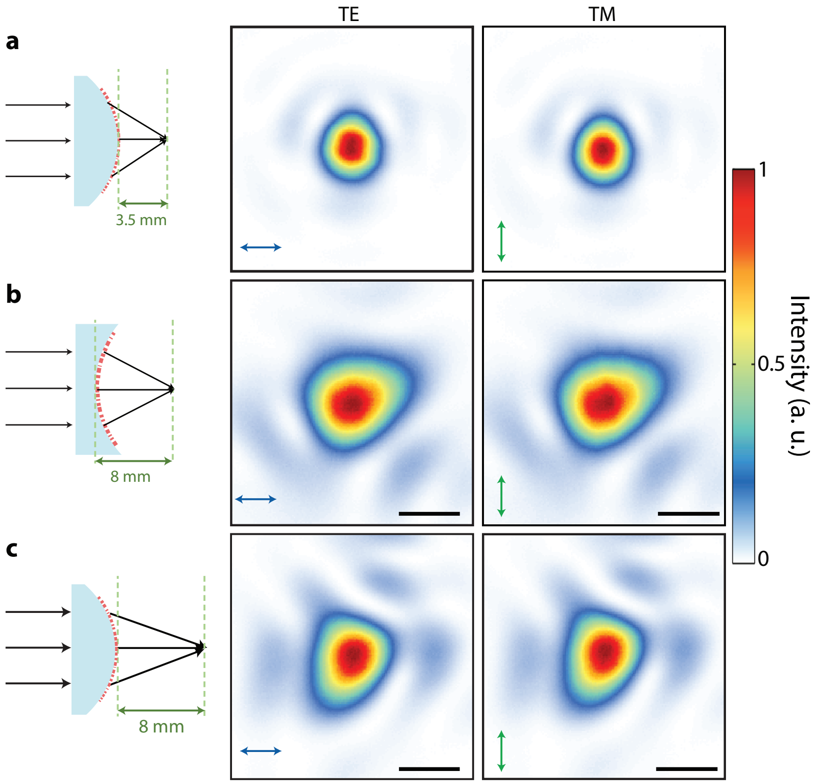

Supplementary Figure 6. **Effect of the input beam polarization on device performance.** Intensity pattern measured at the focal plane of three different conformal metasurfaces with TE (left) and TM (right) polarizations. **(a)** A 1 mm-diameter metasurface on a convex cylinder with a 3.5 mm focal distance, **(b)** A 2-mm-diameter metasurface on a concave cylinder with an 8 mm focal distance, and **(c)** A 2-mm-diameter metasurface on a convex cylinder with an 8 mm focal distance. Focus pattern shows very negligible polarization dependence for all of the devices. The measured efficiencies, however, are more sensitive to polarization. The 1-mm-diameter metasurface with a maximum beam incident angle of 7° has an efficiency of 56% for both polarizations, while for the 2-mm-diameter metasurface lens on convex cylinder with maximum incident angle of 14° the efficiency drops from 68% for TE polarization to 64% for TM polarization. Besides, the metasurface lens on the concave cylinder with a maximum beam incident angle of 9° has efficiencies of 52% and 50% for TE and TM polarizations, respectively. This is in accordance with the angular dependence of transmission coefficient as shown in Supplementary Fig. 2. The device efficiency for TM polarization degrades as the angle between metasurface normal and incident beam increases. However, this does not considerably affect the focus shape for any of the devices. Scale bars, 5 μm. TE: transverse electric, TM: transverse magnetic.

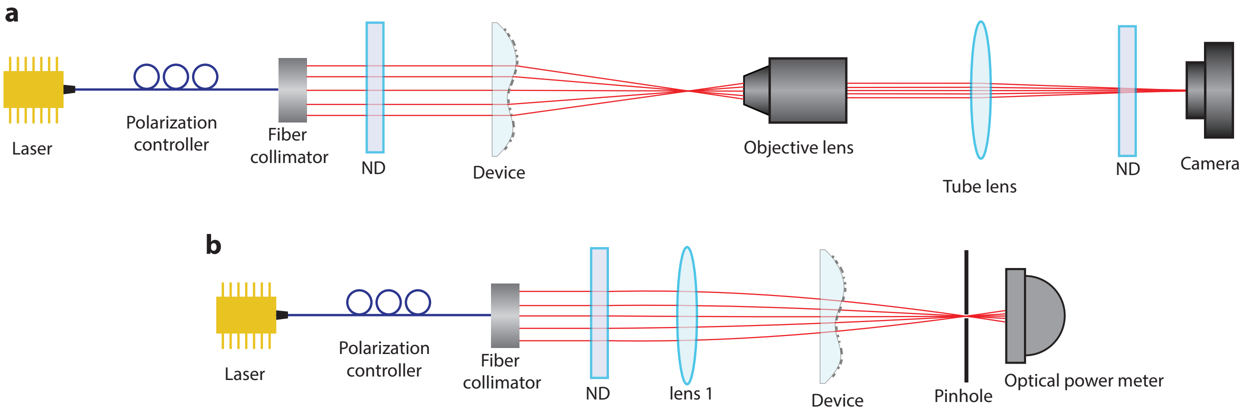

Supplementary Figure 7. **Measurement setup**. **(a)** Schematic illustration of the measurement setup used for characterization of the conformal metasurfaces. ND: neutral density filter. **(b)** Schematic diagram of the experimental setup used for measuring the efficiencies of conformal metasurfaces.

SUPPLEMENTARY NOTE 1: RESONANT MODES OF THE NANO-POSTS

To get more insight into the resonant modes contributing to the transmission coefficient, we consider the amplitude and phase of the transmission coefficient of a periodic array of the nano-posts as a function of wavelength around 915 nm (yellow and green solid curves in Supplementary Fig. 1e). The nano-posts diameter was chosen to be 200 nm, in the middle of our parameter scale. The resonant modes of such an array that have resonant frequencies within the desired bandwidth are found using the finite element method in COMSOL Multiphysics. The magnetic energy density for these resonant modes is shown in supplementary Fig. 1b in two horizontal and vertical cross sections indicated in supplementary Fig. 1a. Total transmission of the nano-posts array is determined by the interference between the incident light and these dominant resonant modes. We have reconstructed the amplitude and phase of the transmission coefficient in the bandwidth of interest using these resonant curves. Supplementary Fig. 1d shows the resonant curves with their corresponding amplitudes used in the reconstruction, along with the reconstructed transmission amplitude. Supplementary Fig. 1e compares the actual transmission amplitude and phase of the nano-posts array with the reconstructed ones. Using more resonant modes (with frequencies outside the bandwidth of interest) it is possible to reconstruct the transmission with even higher accuracy.

SUPPLEMENTARY NOTE 2: ANGULAR DEPENDENCE OF TRANSMISSION COEFFICIENT FOR TE AND TM POLARIZATIONS

Supplementary Fig. 2a shows a schematic illustration of a uniform array of nano-posts embedded in PDMS (left), and its simulated transmission amplitude (top) and phase (bottom) as a function of nano-posts diameter and incident beam angle for TE polarization. Similar plots for TM polarization are shown in Supplementary Fig. 2b. These results show weak angular dependence of the high contrast transmitarray metasurfaces for TE polarization in the range of angles involved in their operation. The larger angular dependence for the TM polarization results in slight degradation of the device performance in this polarization. The maximum angle between the metasurface normal and the incident beam for the two metasurfaces discussed in the main text are 7° and 9°. The angular dependence increases for larger incident angles, indicating that angular dependence should be considered in the design of conformal metasurfaces with steep local incident angles. Moreover, for the lattice used here, higher diffraction orders are present for incident angles larger than $\sim$25°, and a smaller lattice constant should be used for metasurfaces with larger incident angles. Magnetic energy density distributions for a uniform nano-posts array with the diameter of 258 nm illuminated with a plane wave with incident angle of θ =9° for TE and TM polarizations are shown in Supplementary Fig. 2c and 2e, respectively. The corresponding diameter/angle points are indicated by solid squares on the transmission plots presented in Supplementary Figs. 2a and 2b. Similar plots of the magnetic energy density distributions for an incident angle θ=1° are shown in Supplementary Figs. 2d and 2f (indicated by solid circles on the transmission plots in Supplementary Figs. 2a and 2b). The magnetic energy density of the incident beam is chosen to be 1. For obliquely incident beams the symmetry between the TE and TM waves is broken, and this results in various modes in the nano-posts being excited with different amplitudes for the two polarizations. This in turn results in dissimilar behavior for the TE and TM polarizations as a function of the incident angle. Comparing Supplementary Fig. 2c and 2d, we can observe that the magnetic energy density inside the nano-posts is very similar for TE wave with different incident angles. From supplementary Fig. 2e and 2f, however, we can see that the magnetic energy density distribution changes significantly with incident angle for TM wave. This change in excited modes of the nano-posts for the TM waves as a function of angle, which is a consequence of the axial component of the electric field becoming important, results in the stronger angular dependence of the TM transmission observed here.

SUPPLEMENTARY NOTE 3: HIGH FIDELITY OF THE NANO-POST TRANSFER PROCESS

To preserve the high efficiency and the diffraction limited optical performance of the metasurfaces through the transfer process to the PDMS substrate, it is essential that a large majority of the nano-posts are transferred, and the gaps between the nano-posts are uniformly filled with PDMS. The efficiency of the metasurface decreases and wavefront aberrations are introduced if some of the nano-posts are not transferred because the portion of the light passing through the areas with missing nano-posts does not undergo the proper phase shift. Air voids between the nano-posts also degrade the efficiency and cause wavefront aberrations by disturbing the near-field optical distributions of the nano-posts which in turn leads to a lower local transmission efficiency and an incorrect phase shift. To verify that all the nano-posts are transferred to the flexible substrate, after the transfer process, the metasurfaces were examined using an optical microscope. We observed that all the nano-posts were successfully transferred as it is shown in Supplementary Fig. 3a, which shows an optical microscope image of a portion of a flexible metasurface. The void-free filling of the gaps between the nano-posts with PDMS was verified using scanning electron microscopy (SEM). A thin layer of gold ($\sim$15 nm) was deposited on the top surface of the flexible metasurface to avoid charge accumulation during SEM imaging. An SEM image of the metasurface taken at a tilt angle of 30 degrees with respect to the metasurface normal is shown in Supplementary Fig. 3b. The cracks seen in this SEM image at the location of the nano-posts are in the gold layer. They did not initially exist and were slowly appeared as the imaged area was exposed to the electron beam and the sample was charged. As Supplementary Fig. 3b shows, no void is present between the nano-posts, and the same conclusion was made when a larger area of the metasurface was inspected with SEM. According to all different SEM images, the yield of the entire fabrication process is more than 99.5$\%$ with a 95$\%$ confidence interval [1].

SUPPLEMENTARY REFERENCES

[1] Sauro, J. & Lewis, J. R., Estimating completion rates from small samples using binomial confidence intervals: comparisons and recommendations. *SAGE Publications* **49**, 2100–2103 (2005).
